# A Screen-Printed Voltammetric Sensor Modified with Electropolymerized Molecularly Imprinted Polymer (eMIP) to Determine Gallic Acid in Non-Alcoholic and Alcoholic Beverages

**DOI:** 10.3390/polym16081076

**Published:** 2024-04-12

**Authors:** Camilla Zanoni, Lucrezia Virginia Dallù, Clementina Costa, Alessandra Cutaia, Giancarla Alberti

**Affiliations:** Department of Chemistry, University of Pavia, Via Taramelli 12, 27100 Pavia, Italy

**Keywords:** gallic acid, polyphenols, electrosynthesized molecularly imprinted polypyrrole, screen-printed electrodes, differential pulse voltammetry, electroanalysis

## Abstract

This paper presents a low-cost disposable sensor for gallic acid (GA) detection in non-alcoholic and alcoholic beverages using a screen-printed cell (SPC) whose working electrode (in graphite) is modified with electrosynthesized molecularly imprinted polypyrrole (eMIP). Our preliminary characterization of the electrochemical process shows that gallic acid (GA) undergoes irreversible oxidation at potentials of about +0.3 V. The peak potential is not affected by the presence of the eMIP film and alcohol percentages (ethanol) up to 20%. The GA determination is based on a differential pulse voltammetry (DPV) analysis leveraging its oxidation peak. The calibration data and the figures of merit of the analytical method (LOD, LOQ, and linear range) are calculated. To validate the feasibility of the sensor’s application for the dosing of GA in real matrices, some non-alcoholic and alcoholic beverages are analyzed. The results are then compared with those reported in the literature and with the total polyphenol content determined by the Folin–Ciocalteu method. In all cases, the concentrations of GA align with those previously found in the literature for the beverages examined. Notably, the values are consistently lower than the total polyphenol content, demonstrating the sensor’s selectivity in discriminating the target molecule from other polyphenols present.

## 1. Introduction

Polyphenols have aroused interest due to their substantial health benefits, such as their anti-inflammatory and antihistaminic effects, protection against cardiovascular diseases, and potential antitumor activities [1].

Gallic acid (GA, 3,4,5-trihydroxy benzoic acid) is a natural polyphenol mainly present in fruits (for example, grapes, cranberries, and bananas) and beverages such as tea and wines. GA is a joint ingredient in herbal supplements, homeopathic remedies, and pharmaceutical products. Thanks to its remarkable antioxidant activities, it inhibits the interaction of radicals with health-benefitting molecules by yielding H-atoms from its phenol functionalities to free radicals [2,3,4]. It is also often employed in electroanalysis as a standard for determining the total polyphenol content, i.e., the antioxidant capacity index of foods [5,6]. Moreover, the quantification of GA is frequently an index of the authenticity of fruit juices or alcoholic beverages [7].

Monitoring GA concentration is crucial, and several analytical methods have been proposed, such as high-performance liquid chromatography (HPLC) [8], capillary electrophoresis [9], diffuse reflectance spectrometry [10], and chemiluminescence [11]. However, some of these techniques involve complex and laborious sample pre-treatment steps before an analysis can be conducted, coupled with expensive instrumentation.

Electrochemical methods are suitable alternatives to overcome these drawbacks since they provide a low-cost, selective, and sensitive way to detect several analytes.

GA can be efficiently determined by electroanalytical methods since it is an electroactive compound undergoing irreversible oxidation. Moreover, thanks to the development of materials science, the modification of electrode surfaces with conductive or molecularly imprinted polymers (MIPs) or metal nanoparticles allows for the selective sensing of GA [12]. Different electrochemical sensors and methods have been proposed, mainly based on modifying classical macroelectrodes, such as glassy carbon, carbon paste, or gold with acrylic molecularly imprinted polymer films [13,14,15]. Despite their good analytical performances, lengthy and expensive electrode surface modifications are required, limiting their use for research purposes.

As an alternative, the present work presents the employment of screen-printed cells whose working electrode is modified by an electropolymerized molecularly imprinted polypyrrole.

In the last decades, screen-printed cells (SPCs) have grown significantly in demand due to the advantages of screen-printing technology in terms of its low price, versatility, and user-friendliness [16]. Moreover, screen-printed electrodes (SPEs) are among the most adaptable devices for on-site sensing due to their rapid response, low power needs, and respectable level of sensitivity [17]. The outstanding adaptability of SPEs is mainly determined by the numerous methods by which the electrodes’ surface can be modified. The inks’ composition and the electrodes’ surface can be modified by noble metal nanoparticles, polymers, or bioreceptors [18]. SPEs have been indicated as effective sensor substrates for developing disposable MIP-based devices for in situ and point-of-care testing applications.

MIPs are well-known synthetic receptors that simulate the typical recognition system of their biological counterparts (i.e., enzymes for substrates or antibodies for antigens) [19]. Molecular imprinting polymerization is based on a three-reagent synthesis comprising functional monomers, a crosslinker, and the target analyte, the latter acting as a template. After the polymerization, the analyte/template is removed, and specific recognition cavities are formed inside the polymer, matching the shape and size of the analyte. Therefore, when contacted with a sample solution, the empty MIP can rebind the target molecule or closely related compounds specifically [20,21,22,23]. MIPs are attractive for their recognition ability, similar to that of natural receptors. They can be prepared for several target analytes and possess a higher level of physical and chemical stability than bioreceptors [24,25].

The surface imprinting method has recently been highlighted as the most convenient way for depositing MIPs directly on SPEs [26,27,28,29,30,31,32,33,34]. Among different strategies, electropolymerization is very efficient since it grants excellent control of the MIP growth on the electrode’s surface; the thickness can be modulated easily and finely by controlling the polymerization conditions [35,36,37].

The most frequently electropolymerized monomers are pyrrole, aniline, and thiophene derivatives [38,39]. Polypyrrole has often been employed thanks to its water solubility, good stability, conductivity, and redox properties [40].

An electrochemical procedure that can be applied to improve electrosynthesized molecularly imprinted polypyrroles is overoxidation, which is obtained by applying to the MIP film an anodic potential more positive than that necessary for polymerization. The overoxidation is helpful since it allows for the formation of oxygen-containing groups, such as hydroxyl, carbonyl, and carboxyl, which can form hydrogen bonds that can be involved in various electrostatic interactions with template molecules, promoting the formation of highly selective cavities [39]. Sometimes, overoxidation can be used to improve the template removal or the renovation of the sensor’s performance after measurements have been taken [35].

In this scenario, a voltammetric sensor for gallic acid detection is developed and tested to determine the analyte in non-alcoholic and alcoholic beverages. In particular, a molecularly imprinted overoxidized polypyrrole film was electrosinthesized over the working electrode surface (in graphite ink) of screen-printed cells using GA as the template. Differential pulse voltammetry (DPV) is used for our quantitative analysis. Interference tests and an analysis of tea and wine samples were performed to evaluate the selectivity and applicability of the proposed sensor.

Compared to already-presented MIP-modified electrodes for GA detection [13,14,15], the advantages of our sensor are its cheaper electrodes and apparatus, the reduced quantity of reagents and solvents required, the lack of a pre-treatment step for samples, and the possibility of in situ or online analysis thanks to portable instrumentation.

## 2. Materials and Methods

### 2.1. Reagents and Instruments

Pyrrole solution (98%, Merk Life Science S.r.l., Milan, Italy) was purified by a Hickman distillation head until a transparent solution was obtained and it was stored in darkness at 4 °C. Gallic acid (97.5–102.5% titration), lithium perchlorate (LiClO_4_, purum p.a., ≥98.0%), sodium chloride (NaCl, ACS reagent, 99.5%, catechin (ACS reagent, ≥98.0%)), L-ascorbic acid (analytical standard), 2-furaldheyde (ACS reagent, ≥98.0), L-tartaric acid (ACS reagent, ≥99.0%), Folin–Cicolteau reagent, and ethanol (for analysis, ACS reagent), from Merk Life Science S.r.l., Milan, Italy were used as received.

For electrode surface characterization, solutions of potassium hexacyanoferrate(III), potassium chloride, and sodium chloride (Merk Life Science S.r.l., Milan, Italy) were used.

“Tavernello, cantine Caviro 2020” (10.5% white wine), “Tavernello cantine Caviro 2020” (11.5% red wine), “Belvento Velarosa 2022” (13.5% rosè wine), “Giacomo Sperone” (17% marsala wine), and “Lipton” green tea were acquired from a local supermarket (Pavia, Italy).

Screen-printed cells (SPCs) with working and counter electrodes in graphite and a pseudo-reference by Ag/AgCl-ink (Topflight Italia S.P.A., Vidigulfo, Pavia, Italy) were used.

The potentiostat/galvanostat EmStat4s (PalmSens BV, Houten, The Netherlands) was employed for the voltammetric and EIS (electrochemical impedance spectroscopy) measurements.

### 2.2. Modification of SPCs by eMIP and eNIP

Before modification, the SPCs were cleaned with ethyl alcohol and dried under a hood at room temperature.

The eMIP-based electrode was obtained by electropolymerization on the surface of the cleaned working electrode of the SPC by five cycles of cyclic voltammetry (CV), scanning the potential from −0.6 V to 0.8 V at a scan speed 0.1 V/s, in an aqueous solution of 0.1 M LiClO_4_, 15 mM pyrrole, and 0.1 M gallic acid. The overoxidation of the molecularly imprinted polypyrrole film was achieved by chronopotentiometry at a potential of +1.2 V for 120 s in 10 mL of 0.1 M lithium perchlorate solution.

To eliminate the template from the eMIP, a two-step procedure was used: washing it in ethanol for 20 min, followed by 20 cyclic voltammetry scans from −1 to +1 V (with a scan speed of 0.1 V/s) in 0.1 M lithium perchlorate solution at pH of 3 to remove the entrapped template entirely, i.e., until the oxidation peak of gallic acid disappeared.

Electropolymerized, not-imprinted polymer (e-NIP) was prepared following the same procedure but without including gallic acid (template) in the polymerization mixture.

### 2.3. Voltammetric and Amperometric Measurements

The electroactive surface, before and after modifying the electrodes with the eMIP or eNIP, was measured from the parameters of the cyclic voltammetry scans in a probe solution of 5 mM K_3_Fe(CN)_6_/0.1 M KCl at pH of 7, in a potential range −1 V ÷ +1 V, and with different scan speeds from 0.025 to 0.5 V/s.

The intensity of the cathodic or anodic peak was registered and plotted vs. the square root of the scan rate; the effective area was calculated from the slope (*K*) of the obtained straight line and by applying the following equation [41,42]:

(1) $$A=\frac{K}{2.69\cdot 10^{5}\cdot C^{*}\cdot n^{3/2}\cdot D^{1/2}}$$

*n* is the number of exchanged electrons (for K_3_Fe(CN)_6_ *n* = 1), *D* is the diffusion coefficient (for K_3_Fe(CN)_6_, *D* = 3.09 × 10^−6^ cm^2^/s), and C^*^ is the concentration (5 mM) of the electrochemical probe.

The double-layer capacitance [42,43,44] before and after modification of the electrodes was measured by performing cyclic voltammetry in 0.1 M NaCl solution, varying the scan speeds from 0.025 to 0.5 V/s. in a potential interval in which non-Faradaic current is probable, for example, from +0.05 V to −0.05 V. The difference between the cathodic and anodic current values registered at 0.02 V was plotted against the scan speed; the capacitance corresponds to the slope of the straight line obtained. The double-layer capacitance can be obtained by dividing this value by two.

Before and after modification of the electrode surface, electrochemical impedance spectroscopy (EIS) measurements were also taken to characterize the electrode surface further and describe the electrochemical processes at the electrolyte–electrode interface [45,46]. The EIS measurements were taken in 10 mL of a probe solution (in this case, 5 mM K_3_Fe(CN)_6_/0.1 M KCl at pH of 7). The impedance was registered in the 100 kHz–10 mHz frequency range with a sinusoidal potential modulation of 0.05 V superimposed on a DC potential of 0.2 V.

The most known and used electrochemical probes for EIS are K_4_Fe(CN)_6_, K_3_Fe(CN)_6_, and an equimolar mixture of both. Generally, K_4_Fe(CN)_6_ alone is not used since the Warburg effect is too strong and the charge transfer resistance is not well modeled. K_3_Fe(CN)_6_ permits a reasonable resolution of the process at the interface which can be emphasized by changing the concentration of the probe. The mixture of K_4_Fe(CN)_6_/K_3_Fe(CN)_6_ is the most used since it combines the advantages of each probe; for example, in the presence of this mixture, the interface and the diffusion processes can be detected [47]. In the present study, after preliminary measurements using the mixture of K_4_Fe(CN)_6_/K_3_Fe(CN)_6_ followed by measurements of K_3_Fe(CN)_6_ alone, similar results were obtained, so all the subsequent experiments were performed with the last probe.

Gallic acid’s electrochemical oxidation was characterized using cyclic voltammetry and exhaustive coulometry [48,49,50]. In particular, the number of electrons involved in the entire electrochemical process was determined using exhaustive coulometry [48,49,50] by electrolyzing gallic acid at a potential slightly above (from 50 to 100 mV) the oxidation peak in a three-electrode cell under a gentle flow of nitrogen. A Pt gauze was employed as a working electrode, a Pt wire was the counter electrode, and Ag/AgCl/3 M KCl was the reference electrode. The number of electrons was computed from the quantity of charges, *Q* = *I* × *t*, required for the exhaustive electrolyzation of the analyte (i.e., until the current reached 5% of the initial value).

The parameters characterizing the first stage of the oxidation process, i.e., corresponding to the first and well-defined CV wave, were derived from the cyclic voltammograms registered in 10 mL LiClO_4_ 0.1 M/GA 2.5 mM solution at pH of 3 from −1 V to +1 V with different scan speeds from 0.01 to 2 V/s.

The diffusion coefficient *D* (cm^2^/s) was calculated from the slope of the graph intensity of the anodic peak current (*I*_p_, A) vs. square root of the scan rate (*v*, V/s) and by applying Equation (2) as follows, which holds true for irreversible processes [51]:

(2) $$I_{p}=2.99\cdot 10^{5}\cdot n\cdot \sqrt{n_{\alpha }\cdot \alpha_{a}}\cdot A\cdot C^{*}\cdot \sqrt{D}\cdot \sqrt{v}$$

where *n* is the number of exchanged electrons in the first oxidation step, *n_α_* is the number of electrons exchanged in the slowest limiting step (for a one-electron process, *n_α_* = 1), *α*_a_ is the anodic transfer coefficient, A is the active area (cm^2^), C^*^ is the molar concentration of the analyte in the solution, and *D* is the diffusion coefficient (cm^2^/s).

A rough estimate of the formal potential (*E*^0′^) for an irreversible process was obtained from the intercept of the plot anodic peak potential (*E*_p_, V) vs. scan rate (*v*, V/s) [49,52].

The anodic transfer coefficient (*α*_a_, V) for an irreversible process can be calculated by applying Equation (3) as follows [51,53]:

(3) $$\left|E_{p}-E_{p/2}\right|=\frac{1.857\cdot R\cdot T}{\alpha_{a}\cdot n\cdot F}=\frac{47.7}{\alpha_{a}\cdot n}\;\mathrm{mV}\;\mathrm{at}\;25\;{}^{\circ}C$$

where *E*_p_ is the potential of the oxidation peak (V), *E*_p/2_ is the potential value at *i*_p/2_, *R* is the gas constant (J/mol K), T is the temperature (K), *F* is the Faraday constant (C/mol), and *n* is the number of lost electrons.

The charge transfer kinetic constant *k*^0^ was computed by applying Equation (4) as follows [54], knowing *E*^0′^ and *D*:

(4) $$E_{p}=E^{0’}-\frac{R\cdot T}{\alpha_{a}\cdot n\cdot F}\cdot \left[0.78-\ln\frac{k^{0}}{\sqrt{D}}+\ln\sqrt{\frac{\alpha_{a}\cdot n\cdot F\cdot v}{R\cdot T}}\right]$$

where *E*_p_ is the potential of the anodic peak, *R* is the gas constant (J/molK), T is the temperature (K), α_a_ is the charge transfer coefficient (V), *F* is the Faraday constant (C/mol), *D* is the diffusion coefficient (cm^2^/s), *n* is the number of electrons involved in the rate-determining steps, and *v* is the scan rate (V/s).

Gallic acid (GA) was quantitatively determined by differential pulse voltammetry (DPV) in 10 mL of LiClO_4_ 0.1 M at pH of 3 or in hydro-alcoholic solutions with different percentages of ethanol and by applying the following experimental conditions:

*E*_start_ = −1 V; *E*_end_ = 1 V; *E*_step_ = 0.015 V; *E*_pulse_ = 0.02 V; *t*_pulse_ = 0.2 s; and scan rate = 0.05 V/s.

## 3. Results

### 3.1. SPC’s Working Electrode Modification by eMIP and Its Characterization

The molecularly imprinted polypyrrole was prepared using a molar ratio template/functional monomer equal to 1:15, according to previous studies that suggested avoiding molar ratios higher than 1:5, since fewer imprinted sites should be obtained; conversely, molar ratios lower than 1:20 involve a high quantity of templates, which, during the synthesis, can influence the micro-environment of the reaction, hindering the polymerization [55]. As a reasonable compromise between a limited number of recognition cavities (achievable with < five CV scans) and a very thick polymer film with less accessible recognition sites (achievable with > seven to ten CV scans) [56], five CV scans were selected.

The polypyrrole-imprinted film was then overoxidized. Overoxidation involves the formation of ketone groups in the polypyrrole network and disrupts the conjugation without a significant loss of the polymer from the electrode [57]. Moreover, the film thickness tuning is improved, and the background currents are low and stable [36,38,39].

**Figure 1:**
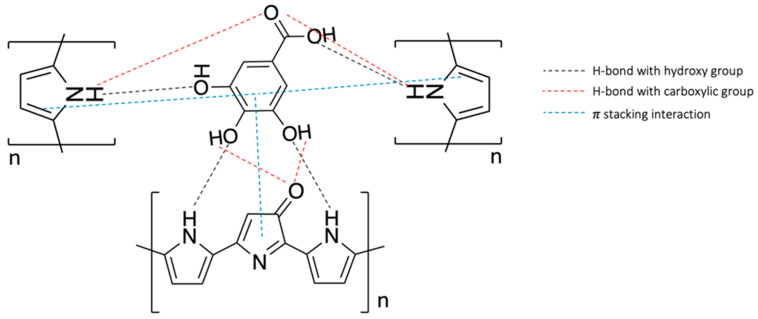
Scheme of the possible interaction mechanism between GA and overoxidate polypyrrole.

In the eMIP-modified electrode, the template molecules are entrapped in the polypyrrole network through non-covalent interactions, i.e., hydrogen bonds between the hydroxyl and carboxylic groups of the gallic acid and the -NH functionalities of the pyrrole units. A scheme of the possible interaction mechanism is shown in Figure 1.

Regarding the electrochemical characterization of the working electrode surface before and after modification with eMIP or eNIP, the electrochemically active area and the double-layer capacitance were determined. Table 1 summarizes our results. Additionally, an electrochemical impedance spectroscopy (EIS) analysis was conducted.

The active area was determined by CV measurements in an electrochemical probe solution (K_3_Fe(CN)_6_) at various scan speeds. The height of the oxidation or reduction current peak was plotted vs. the square root of the scan rate. From the slope of the straight line (K) and by applying the Randles–Sevcik equation (Equation (1)), the active area was computed.

Table 1 shows that the active area decreases after the electrode is coated with a polymer, and, as presumed, the electroactive surface of the eNIP-based electrode is lower than that of the eMIP. Actually, the absence of the recognition cavities in the eNIP leads to a decrease in the electroactive surface.

Regarding the double-layer capacitance, a value of 0.7 (5) μF was obtained for the bare electrode, which is significantly low if compared to that generally achievable with glassy carbon electrodes. This may be due to the different structures of the graphite used as ink for screen printing, in which basal planes prevail instead of the edge planes present in pyrolytic graphite electrodes which show faster electrochemical kinetics, as previously suggested [44]. The double-layer capacitance increased from the bare electrode to the eNIP- and eMIP-modified electrodes (see Table 1); this indicates that the presence of the polymer film increases its ability to gather electrical charges on the electrode’s surface.

Although EIS is a valuable technique for determining the double-layer capacitance for a stable and robust system, it was not applied for this purpose in the present study because screen-printed electrodes suffer from low reproducibility since the surface of each electrode is unique in regards to its impedimetric measurements. Furthermore, if the electrodes are modified with electrosinthesized eMIP, the polymeric film on each electrode can change in morphology and, consequently, capacitance. Conversely, screen-printed electrodes are stable and reliable when used in voltammetric measurements; for this reason, the cyclic voltammetry technique is applied here to determine the double-layer capacitance.

**Figure 2:**
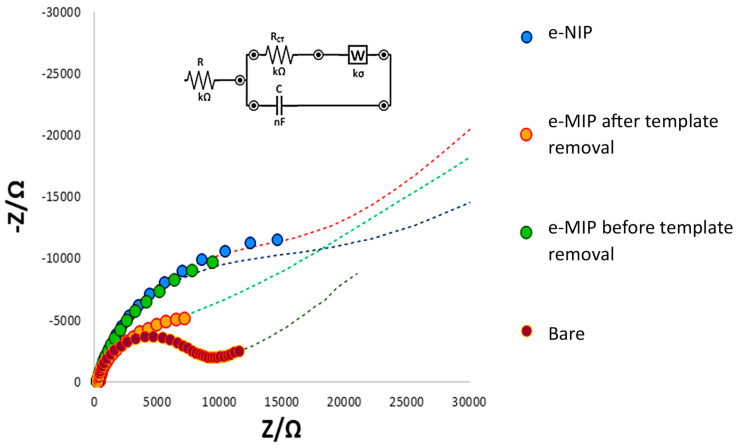
Nyquist plot and Randles equivalent circuit of the bare electrode, eMIP-modified electrode after template removal, eMIP-modified electrode before the template removal, and eNIP-modified electrode. Measurements in 0.1 M KCl/0.05 M K_3_Fe(CN)_6_ electrochemical probe solution.

The bare and modified electrode surfaces were also characterized by EIS measurements. Our results illustrated through the EIS plot may be related to the physico-chemical properties of the electrode’s surface; indeed, the electrochemical responses can be modeled by an equivalent electrical circuit (Randles circuit). Figure 2 shows the Nyquist plot of the bare and modified electrodes (imaginary impedance -Z vs. real impedance Z).

All processes on the bare eMIP- and eNIP-modified electrode surfaces can be modeled by the Randles circuit, reported in the inset in Figure 2. R represents the interface electrode/electrolyte resistance, while R_CT_ is the charge transfer resistance (the diameter of the semi-circle in the Nyquist plot). W is the Warburg element, representing the analyte diffusion in the bulk of the solution, and C is the capacitor at the electrode/electrolyte interface.

The linear part of the Nyquist plot (the straight line at 45°) characteristic for a mass diffusion-limited process appears only for the bare electrode. Moreover, it has the lowest R_CT_ since the graphite ink of the electrode is a good current conductor. The R_CT_ increases if a polymeric film on the working electrode surface is present, which blocks the electrons’ transfer. Since the eMIP features recognition cavities, a lower R_CT_ is recognized for the eMIP-based electrode after template removal compared to that of the same electrode before the elimination of the template that has occupied cavities; indeed, the R_CT_ increases (the diameter of the semicircle increases) by passing from the eMIP after template removal to the eMIP before template removal and finally to the eNIP. Consequently, the diffusion becomes less evident (the linear part of the plot). This behavior is explained by considering that when the analyte molecules are present within the polymer cavities and even more so when the electrode is coated with eNIP, the access of the electrochemical probe to the electrode surface is prevented, thus increasing the resistance to electronic transfer.

Table 2 shows the fitting parameters of the Randles circuital elements schematizing the Nyquist plots of Figure 2.

### 3.2. Characterization of the Gallic Acid Oxidation Reaction at the Bare and eMIP-Modified Electrodes

The electrochemical oxidation reaction of gallic acid was studied using the bare and eMIP-modified electrodes, both in LiClO_4_ 0.1 M solution at a pH of 3 and hydro-alcoholic media containing LiClO_4_ 0.1 M and 20% ethanol, to verify if the polymeric film or the presence of ethanol could affect the process. The effect of the pH was also evaluated only on the bare electrode in the pH range of 2–6; higher pH values were not considered since the polymerization of gallic acid occurs in these conditions, as previously reported [58,59].

**Figure 3:**
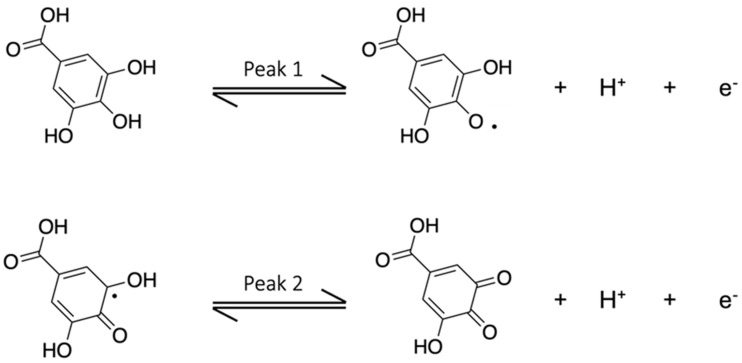
The electrochemical oxidation reaction of gallic acid. (Reproduced with permission from [60], open access Creative Common CC license 4.0, MDPI).

**Figure 4:**
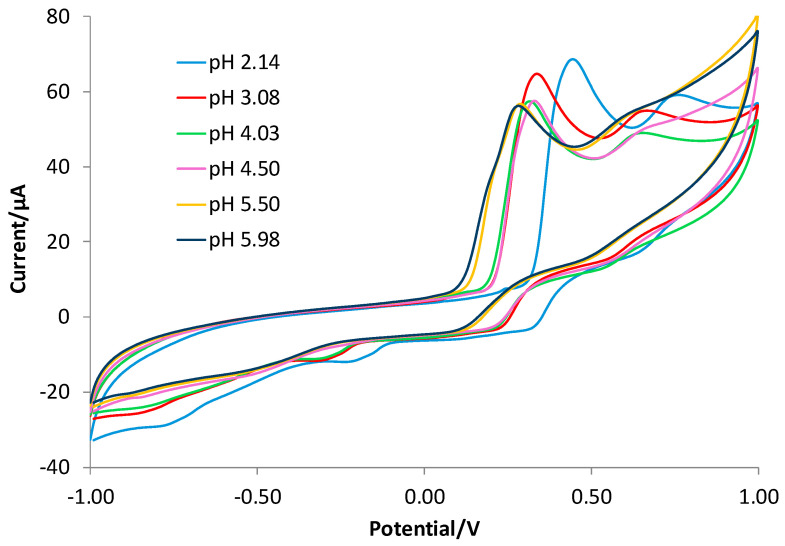
CV registered with the bare electrode in 2.5 mM GA solutions at different pH values. (blue line) LiClO_4_ 0.1 M acidified at pH of 2.14; (red line) LiClO_4_ 0.1 M acidified at pH of 3.08; (green line) LiClO_4_ 0.1 M acidified at pH of 4.03; (violet line) acetate buffer 0.1 M at pH of 4.50; (yellow line) acetate buffer at pH of 5.50; (black line) dihydrogen phosphate 0.1 M at pH of 5.98.

Exhaustive coulometry (EC) was used to evaluate the number of electrons involved in the redox process. The electrode was immersed in 10 mL of 2.5 mM gallic acid solution; the potential was set to +0.7 V and kept fixed for two hours. From the obtained total charge quantity, i.e., 5.08 C, for 25 µmol of electrolyzed GA, it was found that the total number of electrons involved is equal to 2.1, which can be reasonably rounded to 2. It is necessary to observe that the first oxidation peak around + 0.25 V (in the aqueous medium) is the most intense and appreciable one from the CV measurements. The second oxidation peak, which is at more positive potentials (about + 0.6 V in the aqueous solution), is much less intense and is confused with background noise, especially for the measurements taken with the eMIP electrode. This peak is, therefore, unusable for quantitative analysis, as has also been reported by other authors [60]. The following mechanism (Figure 3) can thus be assumed, according to previously published works [60]:

Figure 4 shows the CV graphs obtained with the bare electrode at different pHs.

In Figure 4, the two irreversible waves were observed in the entire pH interval considered (2–6). By decreasing the solution’s acidity, a shift toward less positive potentials occurred for both CV waves, as already verified [59], and the second peak became less clear. Since the aim of the present study was the development of a sensor for GA determination in beverages whose pH is around 3, and also observing from Figure 4 that the higher current value for the first wave is obtained at a pH lower than 4, the following measurements were performed at a pH of 3.

**Figure 5:**
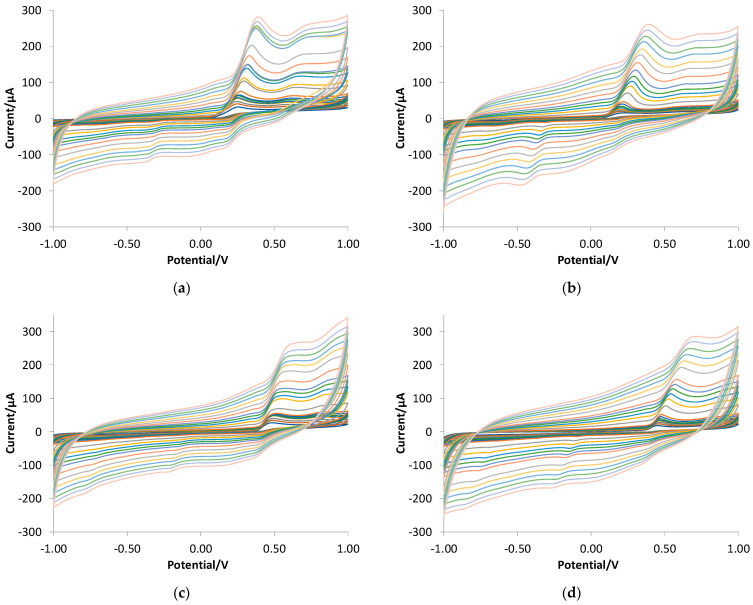
CV registered in 2.5 mM GA solutions at different scan rates (from 0.01 to 2 V/s). (**a**) Bare electrode in LiClO_4_ 0.1 M at pH of 3; (**b**) eMIP-modified electrode in LiClO_4_ 0.1 M at pH of 3; (**c**) bare electrode in LiClO_4_ 0.1 M at pH of 3 + ethanol 20%; (**d**) eMIP-modified electrode in LiClO_4_ 0.1 M at pH of 3 + ethanol 20%.

Figure 5 reports the CV graph obtained with the bare and eMIP-modified electrodes in LiClO_4_ 0.1 M solutions at a pH of 3, with and without ethanol (20%) and at different scan rates, to characterize the first step of the oxidation process (i.e., the peak corresponding to the first and well-defined CV wave). Table 3 summarizes the results.

The linearity of the plot *E*_p_ vs. log *v* suggested that the first stage of GA oxidation follows an EC mechanism, as previously reported [60,61]. The charge transfer coefficient α_a_ is obtained from Equation (3). Acceptable α_a_ values for compounds with irreversible behavior are between 0 and 1. Faster charge transfer processes are characterized by lower values of the charge transfer coefficient, and vice versa for slower charge transfer processes. The results are given in the first column of Table 3. Since the values of α_a_ are all in the range of 0–1, irreversible behavior can be claimed. The values obtained with the bare electrode and the electrode coated with the eMIP are not significantly different, demonstrating that the polymer does not interfere with the electrochemical process. Even in the presence of ethanol, there is no significant change in the α_a_ value.

For an irreversible process, the graph of *E*_p_ vs. the *v* intercept can be considered a rough estimate of the formal potential *E*^0^. The results are shown in Table 3 in the second column. Even in this case, the values obtained with the two electrodes do not differ significantly, as expected, since the eMIP cavities do not interfere with the electrochemical process but are a “preferential way” to access the analyte to the electrode surface. However, the value of *E*^0^ is almost double in the presence of ethanol, which means that the solvent hinders the process. Therefore, it is necessary to apply more positive potentials to cause the oxidation of gallic acid.

The diffusion coefficient *D* can be determined from the slope of the graph of *I* vs. *v*^1/2^ applied to Equation (2). The results are shown in Table 2 in the third column. The *D* values are not significantly different for the bare and eMIP electrodes; the presence of ethanol also does not substantially change the diffusion coefficient of the analyte to the electrode.

Applying Equation (4), the charge transfer kinetic constant *k*^0^ can be derived; the obtained values are given in Table 2 in the fourth column. Even in this case, the values are similar between the bare electrode and that modified with eMIP.

### 3.3. Quantitative Analysis of Gallic Acid by DPV

**Figure 6:**
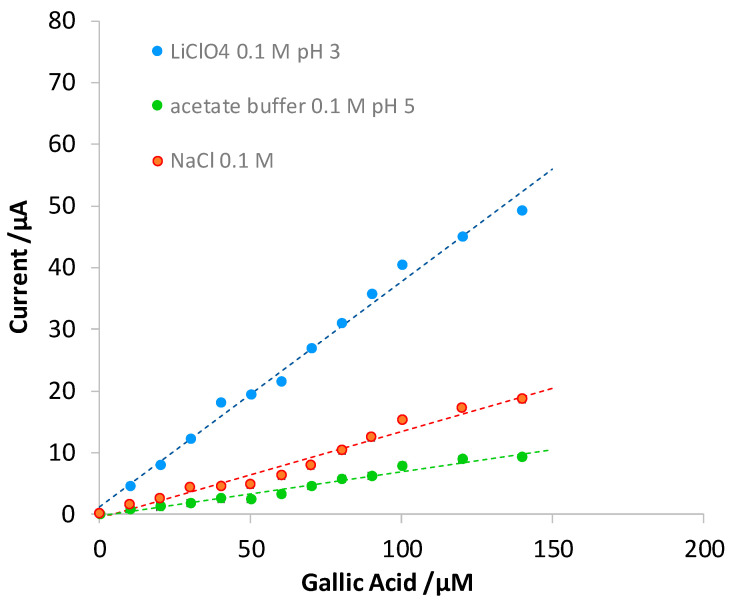
Comparison of three calibration curves in terms of DPV measurements in different ionic media.

Preliminary tests were conducted to identify which ionic medium was the most suitable for developing the voltammetric method for the quantitative determination of gallic acid. Precisely, three different ionic media were compared: acetate buffer 0.1 M at a pH of 4, NaCl 0.1 M, and LiClO_4_ 0.1 M. From the results, it was noted that the analysis is more sensitive when using LiClO_4_ 0.1 M (see Figure 6); moreover, the sensitivity is not affected when acidifying the solution up to a pH of 3.5. It was therefore chosen to work with LiClO_4_ 0.1 M at a pH of about 3–3.5 to simulate the wines’ acidity.

**Figure 7:**
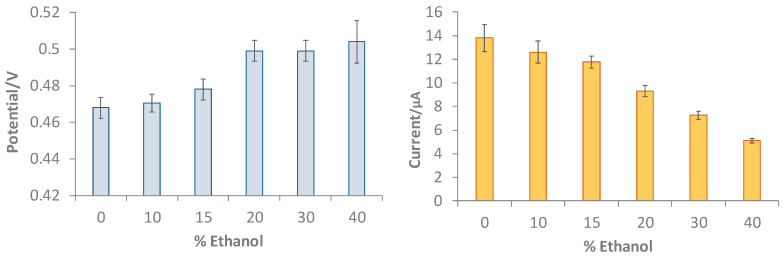
Histograms reporting peak potential (V) and peak current (μa) for 0.2 mM GA solution in LiClO_4_ 0.1 M at pH of 3 containing different percentages of ethanol from 0 to 40%. DPV measurements: potential scan from −1 V to +1 V, potential step of 0.015 V, pulse time of 0.02 s, and scan speed of 0.05 v/s.

To assess the effect of the presence of ethanol on the DPV measurement both in terms of the shift in the peak potential and the value of the current intensity of the anodic peak, measurements were carried out in 0.2 mM gallic acid solution in LiClO_4_ 0.1 M at a pH of 3 containing a percentage of ethanol ranging from 0 to 40%. The results obtained are summarized in the histograms of Figure 7.

The graphs of Figure 7 show that the presence of up to 10% ethanol in the solution does not affect either the potential or peak current. With the increase in the alcohol content, the peak potential progressively shifts, and the current is drastically reduced.

For this reason, it is crucial to quantify gallic acid in beverages, perform calibrations in the appropriate hydroalcoholic medium, and carry out standard addition methods. Since the recorded currents of 40% or more of ethanol are of very low intensity, the voltammetric method is expected to be applied only to beverages with a lower alcohol content; otherwise, a dilution of the sample is needed.

As stated before, the method for quantitative analysis is based on the direct measurement of the analyte using differential pulse voltammetry (DPV), using an oxidation peak at about +0.2 V.

Calibration curves were set up to evaluate the analytical method’s figures of merit (sensitivity, LOD, LOQ, and linear range), while the standard addition method was used to assess the gallic acid content in real samples.

**Figure 8:**
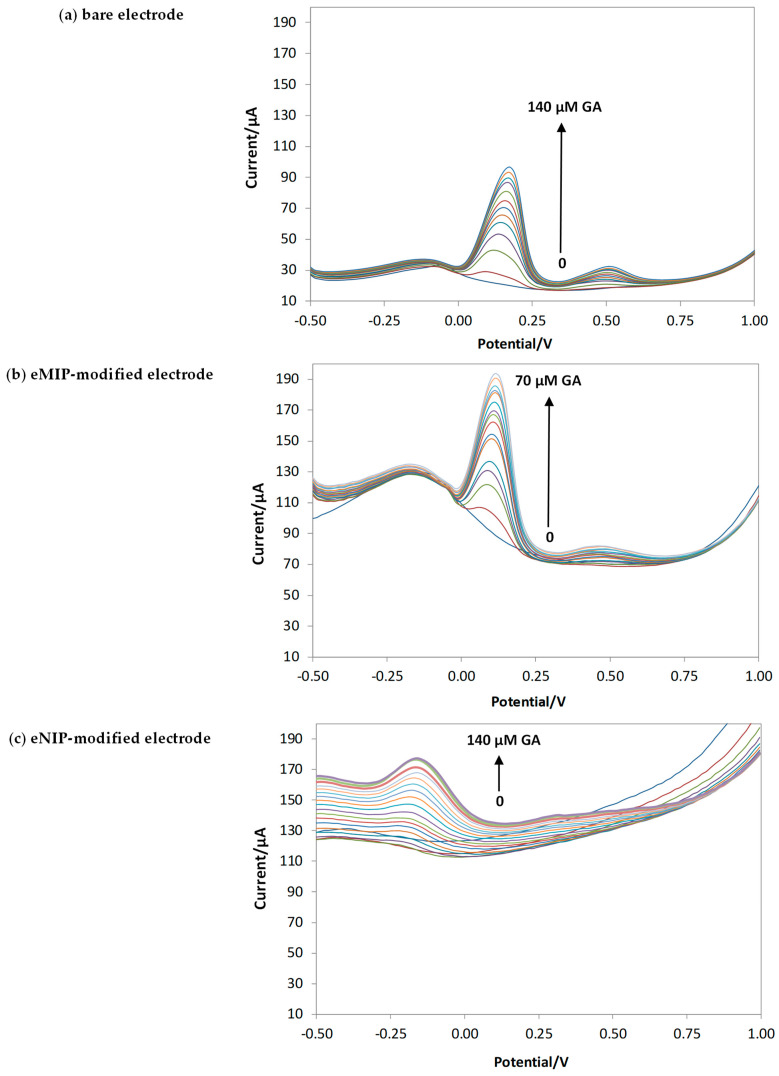
DPV in LiClO_4_ 0.1 M at pH of 3 with increasing concentrations of GA. Potential scan from −1 V to 1 V, potential step of 0.015 V, pulse width of 0.2 V, pulse duration of 0.02 s, and scan speed of 0.05 v/s.

**Figure 9:**
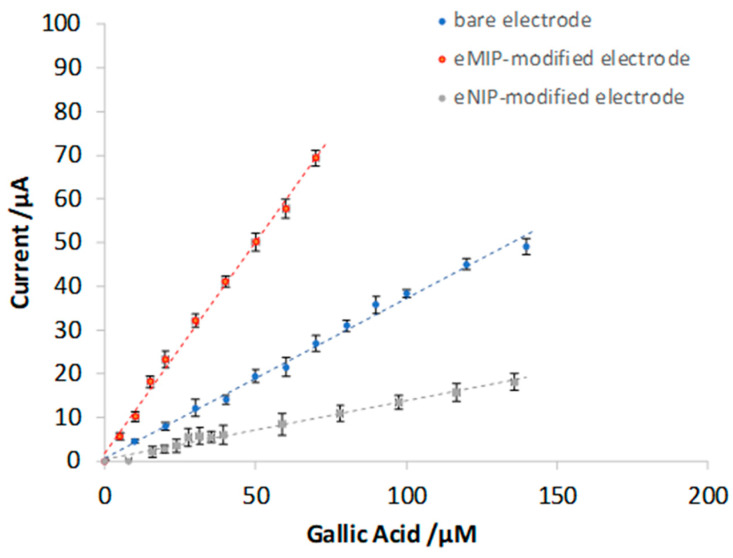
Calibration curves for bare electrodes and eMIP- and eNIP-modified electrodes. The error bars correspond to the standard deviation of the measurements performed with three electrodes of each type.

Figure 8 shows the voltammograms obtained with bare electrodes and eMIP- and eNIP-modified electrodes and Figure 9 reports the calibration curves in the linear range (three replicates for each type of electrode).

Table 4 summarizes the calibration data (the equation of the straight lines) and the analytical method’s figures of merit for the three different electrodes (LOD, LOQ, and linear range).

From the slope of the calibration curve, the LOD and LOQ were computed by applying the following equations:

(5) $$\mathrm{LOD}=\frac{3.3\cdot s_{y/x}}{\mathrm{slope}}$$

(6) $$\mathrm{LOQ}=\frac{10\cdot s_{y/x}}{\mathrm{slope}}$$

where *s_y/x_* is the standard deviation of the y-residuals; this value can be assumed to be the same as the standard deviation of the replicate measurements of blank solutions [62].

The best sensitivity and the lowest LOD are obtained with the electrode modified with eMIP. The slope of the electrode modified with eNIP is much lower, which is desirable since, in this case, the electrode is covered by a polymer film with a low porosity that hinders the arrival of the analyte to the electrode’s surface.

The limit of quantification with the sensor modified by the eMIP equal to about 20 μM corresponds to a GA content of ~3 mg/L. This value affirms the validity of the method for the dosage of gallic acid in alcoholic and non-alcoholic beverages since the amount of GA is much higher in these matrices.

**Figure 10:**
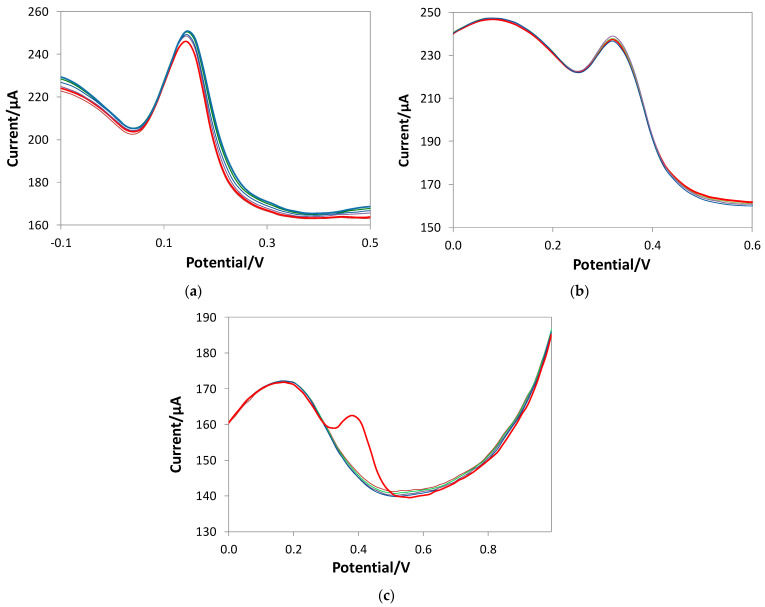
DPV voltammograms recorded in LiClO_4_ 0.1 M at pH of 3 and ethanol 10%, with a fixed GA concentration (red line) and increasing quantities of interferent. DPV measurements: potential scan from −1 V to +1 V, potential step of 0.015 V, pulse width of 0.2 V, pulse duration of 0.02 s, and scan speed of 0.05 v/s. (**a**) Interferent: catechin; (**b**) Interferent: ascorbic acid; (**c**) Interferent: 2-furalhdeyde.

To evaluate the selectivity of the method based on the eMIP-modified sensor, tests were carried out considering three potential interferents: catechin as a different polyphenol, ascorbic acid, a substance used as an antioxidant for food and beverages, and 2-furaldehyde, an oily aldehyde with an aromatic smell and a bitter almond taste. The last molecule is one of the products made by toasting wooden barrels through the conversion of wood sugars and enters the wine during its aging process in the barrique. Catechin and ascorbic acid oxidize at potentials similar to those of gallic acid, while 2-furaldehyde is not electroactive in the potential range used (since it is reduced to a potential of about −0.4 V). Figure 10 shows the voltammograms obtained in solutions containing a fixed concentration of GA (the red line in the voltammograms) and increasing concentrations of the interferent.

The voltammograms obtained show that the eMIP-modified electrode is highly selective in the presence of electroactive and non-electroactive substances under the measurement’s operating conditions. In fact, as can be observed in Figure 10a,b, the addition of catechin or ascorbic acid after registering the voltammogram of gallic acid does not alter the position and the intensity of the peak signal; therefore, the presence of the eMIP film hinders the oxidation reaction of the interfering molecules. The effect of a non-electroactive compound, such as 2-furalhdeyde (Figure 10c), was also considered to verify that the eMIP cavities were not clogged by such molecules, preventing the oxidation of gallic acid. Even in this case, the sensor responded only after the addition of GA to a solution containing up to a ten-times-higher concentration of 2-furalhdeyde.

**Figure 11:**
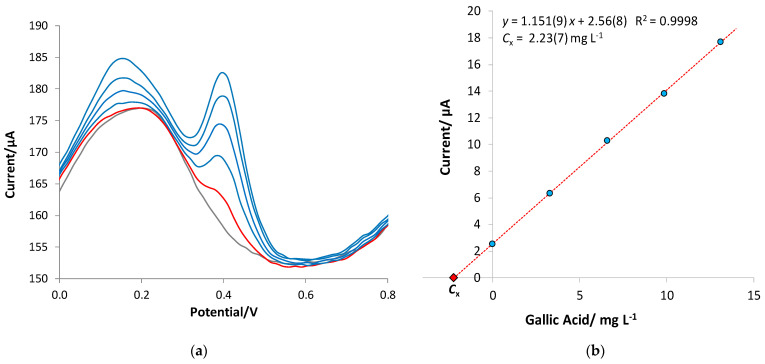
Example of standard addition method applied to a red wine sample. (**a**) DPV voltammograms; and (**b**) standard addition graph of 0.15 mL of red wine diluted with 10 mL of hydroalcoholic solution with 13% ethanol. *C*_x_ = 2.23 (7) mg L^−1^ GA in the diluted sample corresponding to 150 (5) mg L^−1^ GA in the red wine.

To test the feasibility of the eMIP-modified sensor to determine GA in real matrices, some examples of soft drinks (green tea) and alcoholic beverages (white wine, rosé wine, red wine, and marsala wine) were analyzed. The method of standard additions was used for the GA quantification of diluted portions of the samples, using hydroalcoholic solutions as a diluent with the % of ethanol equal to that declared on the product label (three replicates with different dilution ratios). For example, Figure 11 shows the voltammograms and corresponding standard addition graph for a red wine sample.

The total polyphenol content of the same drinks was evaluated using the Folin–Ciocalteau method (expressed as mg/L GAE, gallic acid equivalents).

Table 5 summarizes the results of the analyses and a comparison of our data with the data reported in the literature.

The amount of gallic acid found in all the samples examined is similar to the concentrations previously reported in the literature. The values are always lower than the content of total polyphenols. This experimental evidence further demonstrates the good selectivity of the sensor, which can discriminate the gallic acid from the other polyphenols present in the tested beverages. Obviously, the results must be compared with chromatographic methods such as HPLC-MS to corroborate the data. Only a few samples were analyzed, and we did not compare this technique with classical techniques. In fact, this is the first trial to test the method’s feasibility, and at this stage, we did not intend to proceed with validation.

## 4. Conclusions

In this work, a modified electrochemical sensor for the analysis of gallic acid in non-alcoholic and alcoholic beverages was developed and applied.

The sensor consists of a screen-printed cell for a voltammetric analysis in which the graphite ink working electrode was coated with a molecularly imprinted polymer film obtained by pyrrole electropolymerization in the presence of gallic acid as a template (eMIP). To obtain reproducible measurements with stable and low-intensity background currents, it was necessary to overoxidize the polymer film.

From the preliminary characterization of the electrochemical process, it has been observed that gallic acid irreversibly oxidizes to potentials of about +0.2 V, and the peak potential is not affected by the presence of eMIP film and alcohol percentages (ethanol) up to 10% by volume. Higher alcohol percentages lead to a shift in potential to more positive values and a decrease in current. Therefore, it was concluded that the application of the voltammetric technique for the analysis of alcoholic beverages requires quantification either by a matrix calibration or by the standard addition method. Furthermore, the method is ineffective for quantitative analyses in spirits with an ethanol content of 40% or more; in those cases, dilution of the sample is necessary.

The selected voltammetric method is differential pulse voltammetry (DPV).

We calculated the calibration data and the figures of merit of the analytical method, from which it was verified that the sensitivity of the eMIP-modified sensor is higher than that of the unmodified electrode (bare); moreover, the LOD obtained is sufficient to quantify gallic acid in non-alcoholic and alcoholic beverages.

The feasibility of the sensor’s application for the dosing of GA in real matrices was carried out by analyzing non-alcoholic and alcoholic beverages and comparing the results with those reported in the literature and with the content of total polyphenols determined by the Folin–Ciocalteau method. In all cases, a concentration of gallic acid in line with what was previously found in the literature for the beverages examined was determined, and the values were always lower for the content of total polyphenols. This first trial demonstrates the sensor has a pretty good selectivity and can discriminate the target molecule from other polyphenols present.

## Figures and Tables

**Table 1:** Active area values were calculated using Equation (1). The electrochemical probe solution was 5 mM K_3_Fe(CN)_6_/0.1 M KCl, with a pH of 7.5. Potential scan was from −1 to +1 V; and scan rate was from 0.025 to 0.5 V/s. Double-layer capacitance was measured by CV in 0.1 M NaCl solution. Potential scan was from −0.05 to +0.05 V; and scan rate was from 0.025 to 0.5 V/s.

	Active Area (mm^2^) ^†^	Capacitance (μF)
bare electrode	3.8 (2)	0.7 (5)
eMIP-modified electrode	2.4 (2)	4.11 (3)
eNIP-modified electrode	1.3 (1)	1.49 (3)

^†^ mean values obtained by plotting both the cathodic and the anodic peaks vs. (scan rate)^0.5^; the number in parenthesis is the standard deviation on the last digit.

**Table 2:** Randles circuital elements schematizing the Nyquist plots of Figure 2.

	R (kΩ)	R_CT_ (kΩ)	C (µF)	W (kσ)
bare electrode	0.29	6.5	3.8	4.0
eMIP-modified electrode after template removal	0.51	4.2	77.0	3.1
eMIP-modified electrode before template removal	0.70	10.0	15.4	0.2
eNIP-modified electrode	0.81	15.5	18.8	1.9

**Table 3:** Characterization of the oxidation process at the electrode surface. The parameters are obtained from CV scans (from −1 V to +1 V) at different scan rates (from 0.01 V/s to 2 V/s).

	*α*_a_ (V)	*E*^0′^ (V)	*D* (cm^2^/s)	*k* ^0^
**bare electrode** (LiClO_4_ 0.1M, gallic acid 2.5 mM)	0.67	0.262 (4)	7.4 (4) × 10^−9^	4.9 × 10^−4^
**eMIP-modified electrode** (LiClO_4_ 0.1M, gallic acid 2.5 mM)	0.78	0.216 (6)	4.7 (2) × 10^−9^	4.6 × 10^−4^
**bare electrode** (LiClO_4_ 0.1M, EtOH 20% gallic acid 2.5 mM)	0.59	0.500 (3)	2.9 (6) × 10^−9^	2.7 × 10^−4^
**eMIP-modified electrode** (LiClO_4_ 0.1M, EtOH 20% gallic acid 2.5 mM)	0.79	0.493 (7)	5.0 (2) × 10^−9^	4.7 × 10^−4^

The number in parenthesis is the standard deviation on the last digit.

**Table 4:** Straight line parameters, detection limit (LOD), quantification limit (LOQ), and linear range for bare electrodes and eMIP- and eNIP-modified electrodes. LOD and LOQ have been calculated from the parameters of the lines interpolating the mean values of the measurements of three electrodes for each type (the dotted lines in Figure 6). The number in parenthesis is the standard deviation on the last digit.

Electrode	Slope (µA μM^−1^)	Intercept (µA)	R^2^	LOD (µM)	LOQ (µM)	Linear Range (µM)
Bare	0.37 (1)	0.7 (6)	0.994	12	35	12–140
eMIP	0.97 (2)	1.8 (8)	0.996	5	16	5–70
eNIP	0.134 (5)	0.4 (3)	0.985	18	55	18–140

**Table 5:** Gallic acid concentration in the real samples was analyzed and compared with the total polyphenol content (Folin–Ciocalteau method) and the mean values of gallic acid concentration reported in the literature. Numbers in parentheses are the standard deviations on the last digit.

Beverage	Gallic Acid mg L^−1^	Total Polyphenols GAE, mg L^−1^	Gallic Acid Mean Value, mg L^−1^	Ref.
White wine	14 (4)	181.3	10	[63]
Red wine	145 (15)	1285.1	7.76–172.01	[64]
Rosè wine	120 (13)	1081.1	7.76–172.01	[64]
Marsala	17 (2)	281	13	[65]
Green tea	138 (26)	1535	104	[66]

## Data Availability

Data are contained within the article.
